# Identification and characterization of a novel β-D-galactosidase that releases pyruvylated galactose

**DOI:** 10.1038/s41598-018-30508-4

**Published:** 2018-08-13

**Authors:** Yujiro Higuchi, Hitomi Matsufuji, Masanari Tanuma, Takatoshi Arakawa, Kazuki Mori, Chihaya Yamada, Risa Shofia, Emiko Matsunaga, Kosuke Tashiro, Shinya Fushinobu, Kaoru Takegawa

**Affiliations:** 1 Department of Bioscience and Biotechnology, Faculty of Agriculture, Kyushu University, 6-10-1 Hakozaki, Fukuoka, 812-8581 Japan; 2 Department of Biotechnology, The University of Tokyo, 1-1-1 Yayoi, Bunkyo-ku, Tokyo 113-8657 Japan

**Keywords:** Enzymes, X-ray crystallography

## Abstract

Pyruvyl modification of oligosaccharides is widely seen in both prokaryotes and eukaryotes. Although the biosynthetic mechanisms of pyruvylation have been investigated, enzymes that metabolize and degrade pyruvylated oligosaccharides are not well known. Here, we searched for a pyruvylated galactose (PvGal)-releasing enzyme by screening soil samples. We identified a *Bacillus* strain, as confirmed by the 16S ribosomal RNA gene analysis, that exhibited PvGal-ase activity toward *p*-nitrophenyl-β-D-pyruvylated galactopyranose (*p*NP-β-D-PvGal). Draft genome sequencing of this strain, named HMA207, identified three candidate genes encoding potential PvGal-ases, among which only the recombinant protein encoded by ORF1119 exhibited PvGal-ase activity. Although ORF1119 protein displayed broad substrate specificity for *p*NP sugars, *p*NP-β-D-PvGal was the most favorable substrate. The optimum pH for the ORF1119 PvGal-ase was determined as 7.5. A BLAST search suggested that ORF1119 homologs exist widely in bacteria. Among two homologs tested, BglC from *Clostridium* but not BglH from *Bacillus* showed PvGal-ase activity. Crystal structural analysis together with point mutation analysis revealed crucial amino acids for PvGal-ase activity. Moreover, ORF1119 protein catalyzed the hydrolysis of PvGal from galactomannan of *Schizosaccharomyces pombe*, suggesting that natural polysaccharides might be substrates of the PvGal-ase. This novel PvGal-catalyzing enzyme might be useful for glycoengineering projects to produce new oligosaccharide structures.

## Introduction

*N*-linked protein glycosylation is crucial for several physiological processes, such as protein interaction and cell-cell communication^1–3^. The terminal moiety of *N*-glycans varies in different organisms. Mammalian cells harbor sialic acid attached to the non-reducing end of oligosaccharides^4,5^, whereas the model fission yeast *Schizosaccharomyces pombe* contains pyruvic acid (Pv) at the terminus of cell surface glycans^6–9^. Pyruvylation has been seen in both prokaryotes and eukaryotes. In *Escherichia coli*, for example, Pv is attached to galactose (Gal) moieties of the terminus of colanic acid in capsule structures^10^. *Xanthomonas campestris* produces a heteropolysaccharide xanthan, in which some terminal mannosyl residues are pyruvylated^11^. In extracellular polysaccharides of *Rhizobium leguminosarum* bv. *viciae*-*Pisum sativum*, Pv is attached to *O*-acetylated Gal or glucose (Glc)^12^. There are fewer reports about pyruvylation of oligosaccharides in eukaryotic cells. Pyruvylation of Gal (PvGal) leads to a negative charges on the cell surface, which is important for intercellular interactions in the marine sponge *Microciona prolifera*^13^ and in *S. pombe*^14,15^. The red seaweeds *Cryptonemia seminervis* and *Laurencia filiformis* also contain Pv in their polysaccharide structures^16,17^.

The *S. pombe* pyruvyltransferase Pvg1p has been enzymatically analyzed and found to have substrate specificity for β-linked Gal residues^18^. The *pvg1*^+^ disruptant exhibits less negative charge on its cell surface, owing to a lack of Pv on oligosaccharides of the cell wall. Pvg1p is localized to the Golgi membrane and the substrate phosphoenolpyruvate (PEP) is transported from the cytoplasm to the Golgi by the PEP transporters Pet1p and Pet2p^19^. To elucidate the Pv transfer mechanism of Pvg1p, we previously determined the crystal structure of Pvg1p and derived the structural basis of its substrate specificity^20^.

While the molecular mechanisms underlying the biosynthesis of Pv-oligosaccharides have been relatively well analyzed, enzymes that metabolize and degrade Pv-containing sugar chains remain to be identified. As described above, there are several reports about the existence of PvGal in bacteria; here, therefore, we screened soil samples for microorganisms that exhibit PvGal-ase activity to release PvGal. As a result, we identified a novel gene that encodes a PvGal-ase and characterized its enzymatic activity.

## Materials and Methods

### Microbial culture and microscopy

Soil samples were collected in Yame city, Fukuoka prefecture, Japan. LB medium (0.5% yeast extract, 1% tryptone, 1% NaCl and 3% agar, pH 7.0) was used to culture bacteria on plates or in liquid at 30 °C, with shaking at 200 rpm. Cells of the isolated bacterial strain were observed by using an Eclipse 80i microscope (Nikon), equipped with a Plan Apo 100x/1.40 NA oil objective lens (Nikon), a CoolSNAP EZ CCD camera (Photometrics) and the software MetaVue (Molecular Devices). *S. pombe* wild-type ARC039 (*h*^*−*^ *leu1-32 ura4-C190T*) and *pvg1*Δ strains were grown in YES liquid medium (0.5% yeast extract, 3% glucose, supplements) at 30 °C, with shaking at 200 rpm.

### Preparation of p-nitrophenyl-β-D-pyruvylated galactopyranose

*p*-nitrophenyl-β-D-pyruvylated galactopyranose (*p*NP-β-D-PvGal) was prepared as described previously^18^. Briefly, 0.5 mg of recombinant fission yeast pyruvyltransferase Pvg1p was incubated with 40 mM PEP monopotassium salt and 1 mg of *p*NP-β-Gal in 150 µl of 0.2 M MOPS-NaOH buffer (pH 7.5) at 37 °C overnight. *p*NP-β-D-PvGal was collected by HPLC using a GL-7400 system equipped with a UV spectrophotometer (GL sciences) and a Cosmosil 5C18 reverse-phase column (Nacalai tesque, 4.6 × 150 nm) set at 30 °C. The sample was separated with the developing solvent (0.3% ammonium acetate, pH 5.0, 8% acetonitrile) at a flow rate of 1 ml/min and the eluate was detected at 265 nm.

### Enzymatic analysis

PvGal-ase activity was determined by using *p*NP-β-D-PvGal as a substrate. To screen for microbes with PvGal-ase activity, 5 μL of culture supernatant was mixed with 4 mM substrate in 10 μL of 50 mM acetate buffer, pH 6.0. To determine substrate specificity, 3 μg of ORF1119 protein was mixed with each *p*NP substrate at 3 mM in 5 μL of 200 mM phosphate buffer, pH 7.5. To determine the optimum pH, 10 μg of ORF1119 protein was mixed with 2 mM *p*NP-β-D-PvGal in 10 μL of 400 mM buffers, which varied in pH from 4.0 to 9.5 in increments of 0.5 as follows; acetate buffer, pH 4.0–6.0; phosphate buffer, pH 6.0–8.0; Tris-HCl buffer, pH 7.0–8.5; and MOPS-NaOH buffer, pH 7.0–9.5. To investigate thermal stability, the enzyme was incubated at 4, 30, 37, 42, 50 or 55 °C for 10 min. After incubation, 10 μL of 1 M NaOH was added to terminate the reaction, and the released *p*NP was assessed from the absorbance at 405 nm. One unit of enzyme activity was defined as the activity producing 1 μmol of *p*NP per min. α-D-Xyl and β-D-Xyl were purchased from Seikagaku; *p*NP-β-D-Glc, *p*NP-β-D-Gal and *p*NP-β-D-Fuc were purchased from Sigma.

### Genomic DNA analysis

Genomic DNA of strain HMA207 was prepared by using the ISOPLANT extraction kit (Wako) in accordance with the manufacturer’s instructions. The 16S rRNA gene sequence was amplified from the extracted genomic DNA sample of strain HMA207 by PCR using the universal primers listed in Supplementary Table 1. The DNA sequence of the PCR product was applied to a BLAST (http://blast.ncbi.nlm.nih.gov/Blast.cgi) search, and the species of strain was determined. Whole-genome shotgun sequencing of strain HMA207 was conducted by using a MiSeq sequencer (Illumina). The program Platanus version 1.2.1 was used for sequence assembling. Both Glimmer version 3.02b and BLAST 2.2.26 were used for annotation of the genome. Search for glycoside hydrolase (GH) families was performed according to the CAZy website (http://www.cazy.org/Glycoside-Hydrolases.html).

### Preparation of recombinant proteins

To prepare recombinant expression plasmids, three candidate PvGal-ases, BglC and BglH genes were amplified by PCR using the DNA polymerase PrimeStarGXL (Takara), primers listed in Supplementary Table 1, and genomic DNA of HMA207, *Clostridium saccharoperbutylacetonicum* and *Bacillus subtilis* as a template, respectively. The amplified DNA was ligated into the pET-32b vector, which incorporated a His_5_ sequence at the N-terminus, by using an In-Fusion HD Cloning Kit (Takara). *Escherichia coli* BL21(DE3)CodonPlusΔ*lacZ* strain harboring each candidate PvGal-ase expression plasmid was precultured in 3 ml of LB medium at 30 °C overnight. The OD_600_ was adjusted to 0.05 and the cells were cultured until OD_600_ = 0.5. Next, 100 mM IPTG was added and the cells were cultured at 160 rpm and 15 °C for 48 h. Cells were lysed by ultrasonication on ice and the cell lysate was centrifuged at 20600 × *g* for 10 min at 4 °C. To purify the recombinant protein, the resultant supernatant was applied to a HisTrap^TM^ FF 1 mL column (GE Healthcare) in accordance with the manufacturer’s instructions.

### Preparation of mutant enzymes

Mutations into ORF1119 protein were introduced by PCR using the PrimeSTAR mutagenesis Basal Kit (Takara), primers listed in Supplementary Table 1, and the pET-32b-based expression plasmid as a template. The mutated genes were confirmed by DNA sequencing (Macrogen, Japan) to ensure that only the desired mutations were introduced. The mutants were expressed and purified by using a procedure similar to that described for the wild-type ORF1119 enzyme.

### TLC analysis

The purified ORF1119 protein (3 μg) was incubated with each *p*NP sugar at 3 mM in 5 μL of 200 mM phosphate buffer (pH 7.5) at 37 °C overnight. The reaction samples were separated by TLC by using a TLC Silica gel 60 plate (Millipore) and 1-butanol/ethanol/water (2:1:1, v/v/v) solvent. The TLC plate was sprayed with 0.2% orcinol and 10% methanol/sulfuric acid, baked at 120 °C, and visualized for spots.

### Crystallography

For crystallization trials, immobilized metal chromatography-purified protein was concentrated and further purified by gel filtration chromatography using a Superdex 200 pg 16/60 column (GE Healthcare, Fairfield, CT) in 50 mM HEPES-NaOH (pH 7.4) containing 150 mM NaCl. Ligand-free crystals were obtained at 20 °C using the sitting drop vapor diffusion method. A 0.5-µL aliquot of protein solution containing 12 mg/mL of ORF1119 and 50 mM *p*NP-β-D-PvGal was mixed with an equal volume of a reservoir solution containing 0.1 M phosphate-citrate buffer (pH 5.2) and 50% PEG 300. Despite supplementation of the protein solution with substrate, the crystal structure was obtained as a ligand-free form. Crystals of the E163A mutant complexed with PvGal were obtained by the co-crystallization method. A 0.5-µL aliquot of protein solution containing 17 mg/mL of ORF1119 E163A and 50 mM *p*NP-β-D-PvGal was mixed with an equal volume of a reservoir solution containing 0.1 M phosphate-citrate (pH 5.2), and 32% PEG 300. Crystals appropriate for X-ray diffraction experiments grew in at least 1 week. Crystals were flash-cooled at 100 K in a stream of nitrogen gas. X-ray diffraction data were collected by using a charge-coupled device camera on beamline BL-17A at the Photon Factory of the High Energy Accelerator Research Organization (KEK, Japan) or beamline BL-26B1 at SPring-8 (Japan). The dataset was indexed, integrated, and scaled by using HKL2000^21^. The initial phase was determined by the molecular replacement method using MOLREP^22^. Manual model building and refinement were carried out by using Coot^23^ and Refmac5^24^. A model structure of PvGal was built by using JLigand^25^. Molecular graphics were prepared by using PyMOL (DeLano Scientific, Palo, Alto, CA).

### Kinetic analysis

The kinetic parameters of the wild-type and mutant enzymes of ORF1119 toward *p*NP-β-D-PvGal, *p*NP-β-D-Gal, *p*NP-β-D-Glc and *p*NP-β-D-Fuc were determined by using a discontinuous assay in which the liberation of *p*NP was measured at an absorbance of 405 nm. To determine the kinetic parameters for *p*NP-β-D-PvGal, different concentrations of substrate (1.0–10 mM) and enzyme (S427E, 23 μg; Y436A, 1.6 μg; and others, 0.14–0.54 μg) in 50 mM HEPES-NaOH (pH 7.5) were separately warmed at 37 °C, and the reaction was initiated by mixing the substrate (4.0 μl) and enzyme (6.0 μl) solutions. After an appropriate time interval (typically, from 3 to 12 min), a 2.0 μl aliquot was removed and a 4.0 μl of 1 M Na_2_CO_3_ was added to stop the reaction. The absorbance was measured by using a NanoDrop ND-1000 Spectrophotometer (Thermo Fisher Scientific). To determine the kinetic parameters for the other three substrates, 50 μl of substrate solution (0.25–100 mM) and 75 μl of enzyme solution (0.012–0.54 μg) in 50 mM HEPES-NaOH (pH 7.5) were mixed to initiate the reaction. After an appropriate time interval (typically, 5 to 20 min), a 25 μl aliquot was sampled, and 50 μl of 1 M Na_2_CO_3_ was added to stop the reaction. The absorbance was measured by using a Synergy H1 microplate reader (BioTek).

### Alcian blue staining

To determine the level of extracellular negatively-charged glycans on *S. pombe*, alcian blue staining was performed as described previously^18^. To release Pv from Gal residues in cell wall oligosaccharides, *S. pombe* cells (OD_600_ of 2.0) were suspended in 100 μl of 50 mM phosphate buffer (pH 7.5), incubated with 25 μg of recombinant ORF1119 at 30 °C for 1 hour and subsequently stained with alcian blue.

### Accession numbers

The nucleotide sequences of the ORF1119, ORF4395 and ORF4971 genes have been deposited in the DDBJ/EMBL/GenBank under the accession nos. LC306881, LC306883 and LC306885, respectively. The coordinates and structure factors of ligand-free WT ORF1119 and PvGal-bound ORF1119 E163A mutant have been deposited in the Protein Data Bank under accession codes 5YHS and 5YIF, respectively.

## Results

### Identification of a soil microorganism with PvGal-ase activity

**Figure 1:**
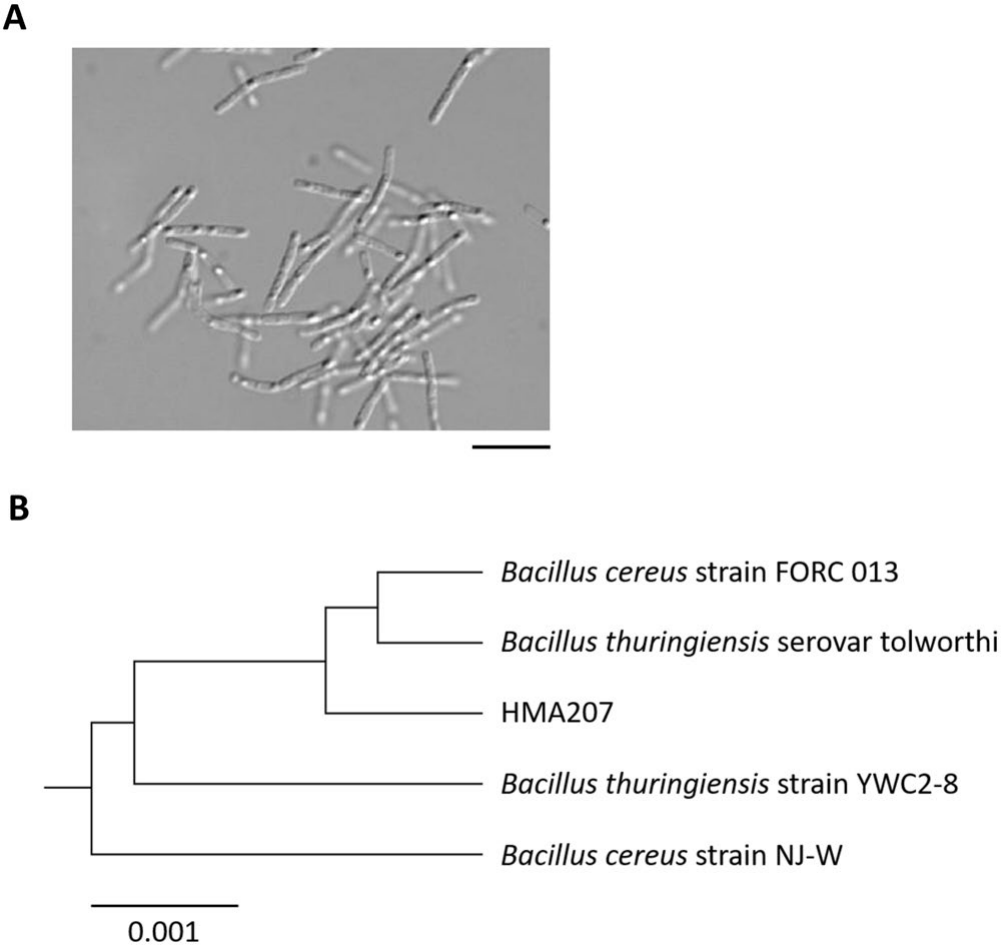
Identification of strain HMA207. (**A**) Microscopic image of strain HMA207. Bar, 10 µm. (**B**) Phylogenetic tree of 16S rRNA gene sequences from *Bacillus* species constructed by using the neighbor-joining method of the program CLUSTAL W (http://www.genome.jp/tools/clustalw/).

To search for a PvGal-ase, we isolated more than 200 bacterial strains from soil samples. Culture supernatants of one isolated strain, named HMA207, exhibited PvGal-ase activity when *p*NP-β-D-PvGal was used as a substrate. HMA207 appeared to be a Gram-positive and bacillary bacterium (Fig. 1A). To identify the strain, we performed a BLAST search based on the 16S rRNA gene sequence, and confirmed that it belongs to the species *Bacillus* (Fig. 1B).

### Exploration of candidate PvGal-ase genes in strain HMA207

Next, to search for genes encoding a potential PvGal-ase, we conducted whole-genome shotgun sequencing of strain HMA207. As a result, 7.78 Gbp were generated from 3.38 × 10^7^ sequencing reads, yielding 1,470 fold-coverage, and 18 contigs were assembled. We determined most of the genome sequence, the details of which will be reported elsewhere. Because *p*NP-β-D-PvGal can be cleaved by β-D-Gal-ase, we searched for β-D-Gal-ase-related genes from the gene annotation generated from our genome analysis; however, no β-D-Gal-ase-related genes were predicted. In addition, we did not find any genes encoding putative GH2 β-galactosidase or GH53 β-galactanase. Thus, among 31 putative glycosidases in the genome of strain HMA207, we selected three genes (ORF1119, ORF4395 and ORF4971) (Table 1). The three genes were selected for the following reasons: ORF1119 is a GH1-like β-D-Gal-ase; and ORF4395 and ORF4971 are predicted to be disaccharidases.

**Table 1:** Candidate genes encoding PvGal-ase in strain HMA207.

ORF	Product^a^	GH^b^	Size (aa)
1119	aryl-phospho-β-D-glucosidase	1	469
4395	diacetylchitobiose-6-phosphate hydrolase	4	441
4971	trehalose-6-phosphate hydrolase	13	553

^a^Based on predictions of BLAST (http://blast.ncbi.nlm.nih.gov/Blast.cgi).

^b^Based on predictions of CAT (http://mothra.ornl.gov/cgi-bin/cat/cat.cgi).

### Enzymatic activities of recombinant ORF1119 protein

**Figure 2:**
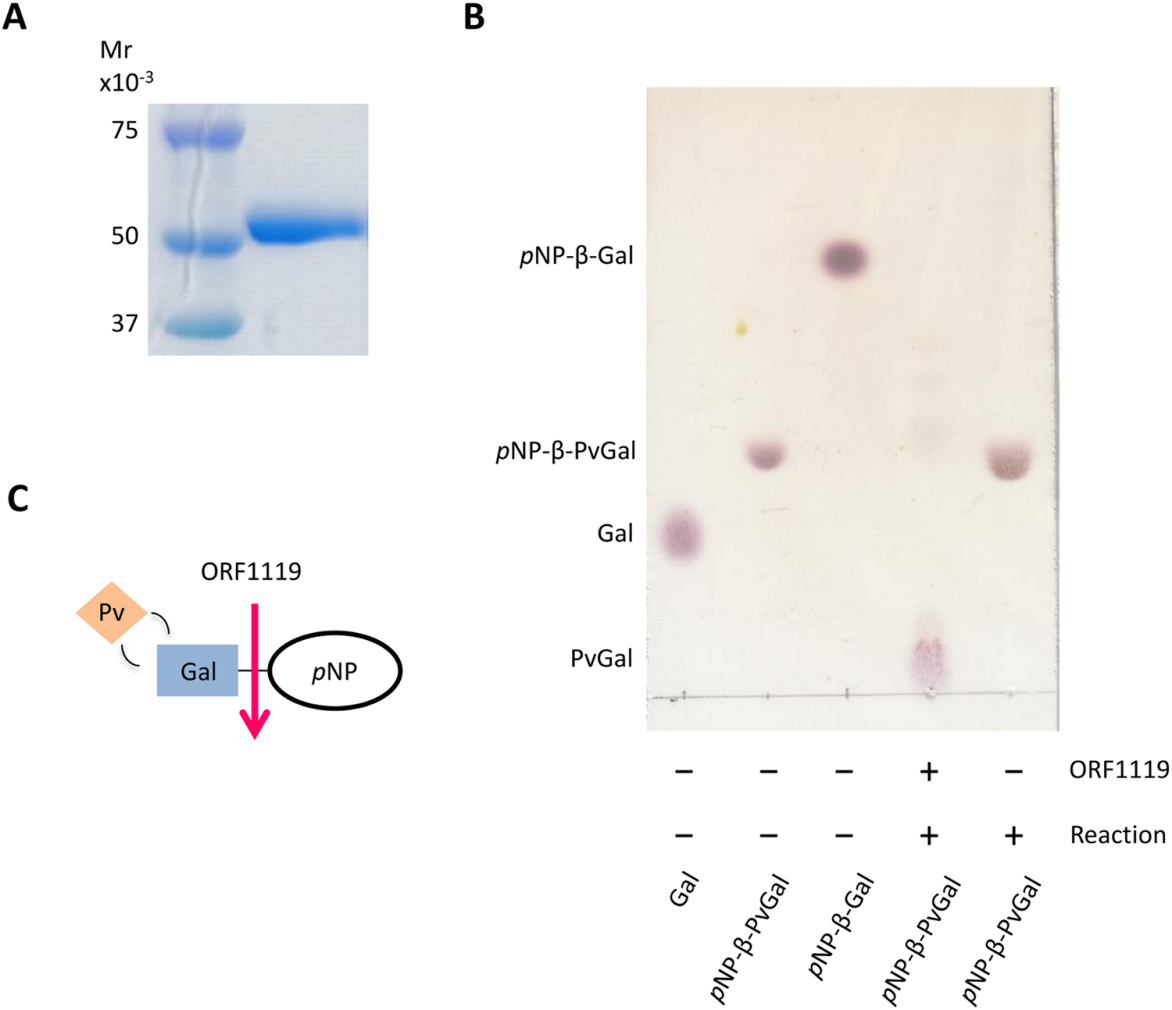
TLC analysis of ORF1119 PvGal-ase activity. (**A**) Purification of recombinant ORF1119 protein. The full-size gel picture is shown in Supplementary Fig. 1. (**B**) TLC analysis of PvGal-ase activity. Galactose (Gal), *p*NP-β-PvGal and *p*NP-β-Gal were spotted as references. The reaction product with ORF1119 protein generated a spot derived from PvGal. (**C**) Schematic diagram of PvGal-ase activity for *p*NP-β-PvGal as a substrate.

We introduced the ORF1119, ORF4395 and ORF4971 sequences into an *E. coli* expression vector lacking *lacZ* to circumvent the potential risk of β-Gal-ase contamination in subsequent enzymatic assays. Crude samples from *E. coli* cells expressing each ORF were tested for PvGal-ase activity using *p*NP-β-D-PvGal as substrate, and found that only ORF1119 exhibited PvGal-ase activity. Amino acid sequence of ORF1119 was predicted to lack signal peptide, suggesting that ORF1119 is an intracellular protein. We prepared recombinant ORF1119 protein and purified it by using a Ni affinity column (Fig. 2A). Using TLC, we then analyzed the reaction between recombinant ORF1119 protein and *p*NP-β-D-PvGal as substrate. The reaction sample showed a spot that was not identical to either *p*NP-β-D-PvGal or *p*NP-β-D-Gal, suggesting that ORF1119 protein cleaves the linkage between Gal and *p*NP to release PvGal (Fig. 2B,C).

### Enzymatic properties of ORF1119 PvGal-ase

**Figure 3:**
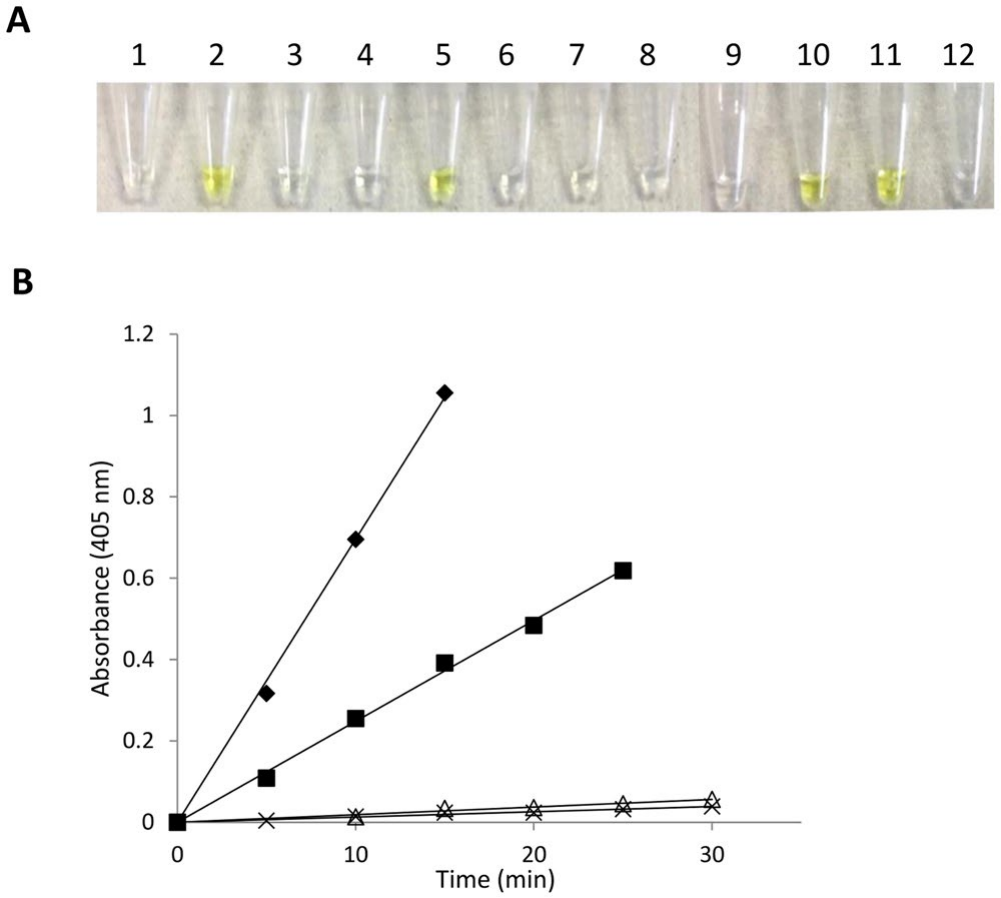
PvGal-ase and other glycosidase activities of ORF1119 protein. (**A**) Substrate specificity of ORF1119 protein: 1, *p*NP-α-Gal; 2, *p*NP-β-Gal; 3, *p*NP-α-Man; 4, *p*NP-β-Man; 5, *p*NP-β-Glc; 6, *p*NP-β-GlcNAc; 7, *p*NP-β-Lac; 8, *p*NP-β-GalNAc; 9, *p*NP-α-Xyl; 10, *p*NP-β-Xyl; 11, *p*NP-β-PvGal; 12, *p*NP-β-PvLac. (**B**) Enzymatic activity toward *p*NP-β-PvGal (rhombus), *p*NP-β-Gal (square), *p*NP-β-Glc (open triangle) and *p*NP-β-Xyl (cross) as substrates.

To determine the substrate specificity of the recombinant ORF1119 protein, we measured its hydrolytic activity over a 1-h reaction time at 37 °C using a variety of *p*NP-glycosides. In addition to *p*NP-β-D-PvGal, ORF1119 exhibited hydrolase activity for *p*NP-β-D-Gal, *p*NP-β-D-Glc and *p*NP-β-D-Xyl (Fig. 3A). To analyze substrate specificity more in detail, we monitored the time course of hydrolytic activity and found that ORF1119 exhibits 2.27 and 1.62 U/mg protein for *p*NP-β-D-PvGal and *p*NP-β-D-Gal, respectively (Fig. 3B). Collectively, ORF1119 protein has highest activity for *p*NP-β-D-PvGal, confirming that this enzyme preferentially hydrolyzes β-D-PvGal.

The optimum pH for ORF1119 PvGal-ase activity was found to be 7.5. Examination of the thermal stability of the enzyme by heating at various temperatures for 10 min indicated that the enzyme was stable at temperatures up to 42 °C.

Next, we examined *K*_m_ values for *p*NP-β-D-PvGal and *p*NP-β-D-Gal, and unexpectedly found that they were 0.81 mM and 3.3 mM, respectively. However, we determined *k*_cat_/*K*_m_ values for *p*NP-β-D-PvGal and *p*NP-β-D-Gal, and revealed that they were 6.3 mM^−1^·s^−1^ and 0.06 mM^−1^·s^−1^, respectively. Taken together, these findings suggest that the stronger activity toward PvGal-ase is due to the high *k*_cat_ value.

### Analysis of ORF1119 homologs

**Figure 4:**
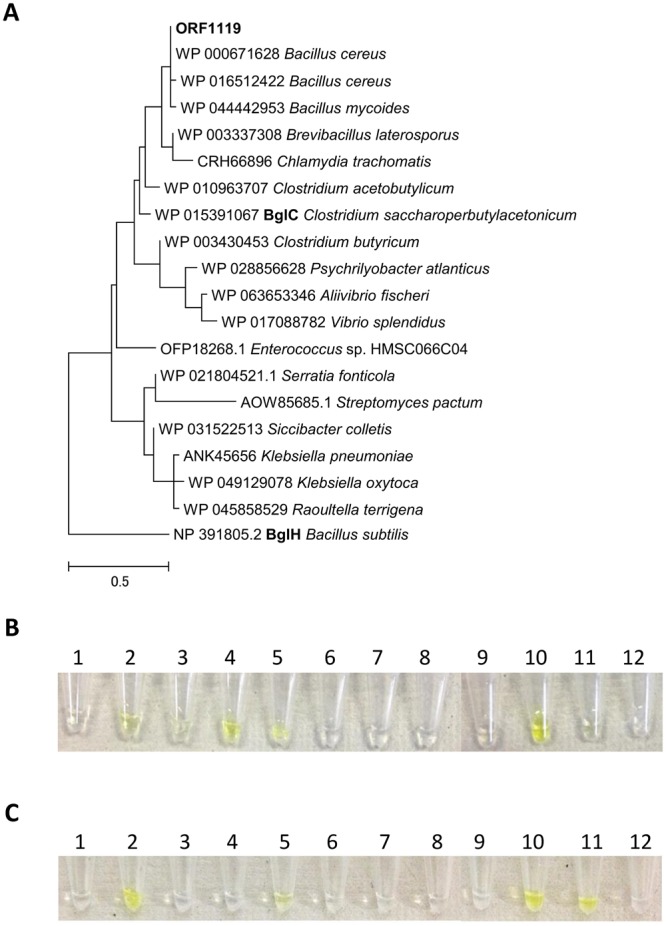
Phylogenic tree of ORF1119 PvGal-ase. The amino acid sequences of ORF1119 homologs were retrieved by a BLAST search and phylogenetically analyzed by using the program CLUSTAL W. The accession numbers are indicated.

**Figure 5:**
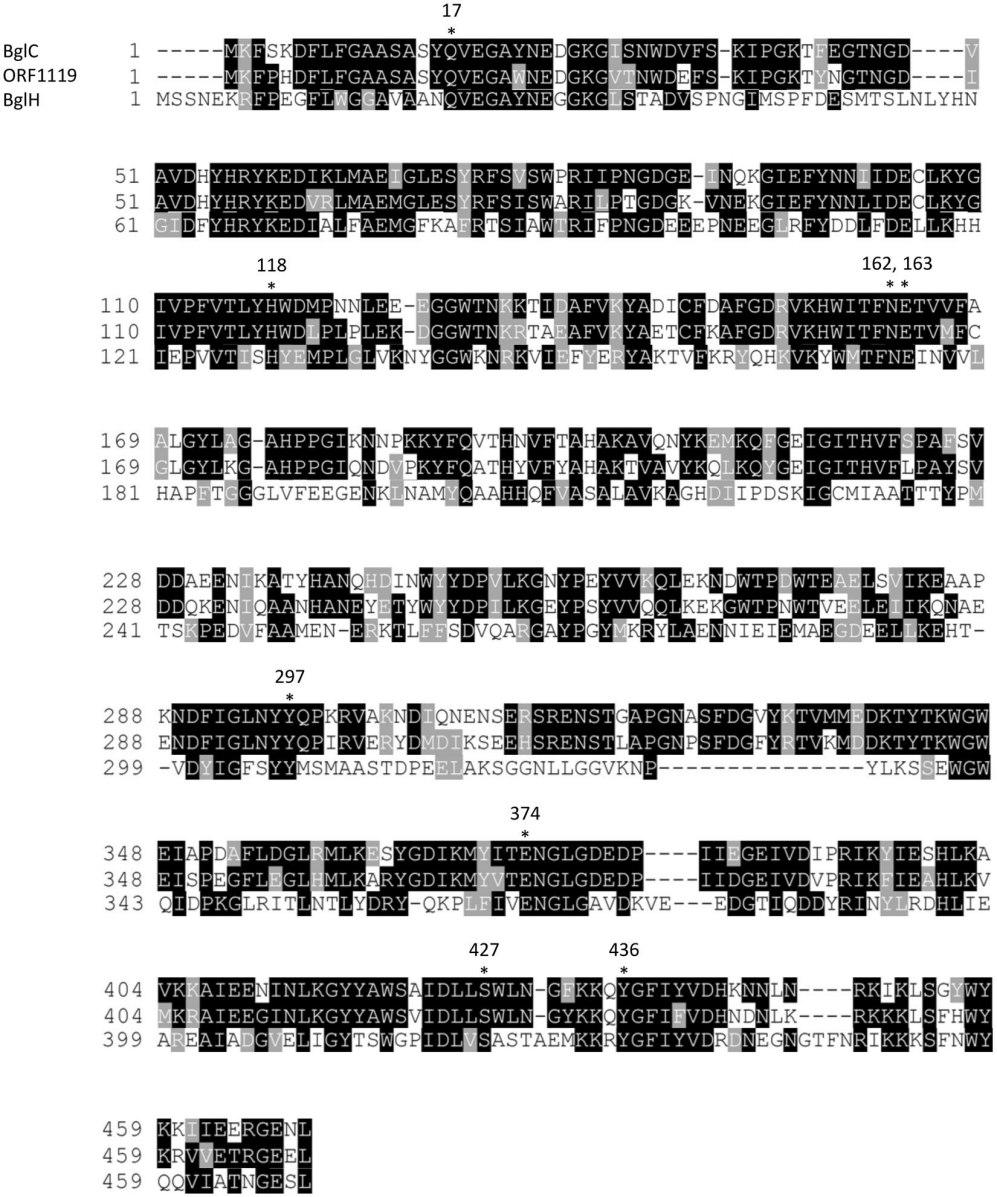
Alignment of ORF1119 PvGal-ase and its homologs. Alignment of the ORF1119 PvGal-ase sequence and its homologs BglC and BglH is shown. Asterisks indicate conserved amino acid residues that are predicted to be crucial for PvGal-ase activity. E163 and E374 are conserved catalytic acid/base and nucleophile residues of GH1.

Next, to obtain information on potential homologs, we performed a BLAST search based on the amino acid sequence of ORF1119 and found that ORF1119 homologs widely exist in bacteria (Fig. 4). Among these homologs, *Bacillus subtilis* BglH belongs to the GH1 family, similar to ORF1119, and exhibits 29.4% identity to ORF1119 (Fig. 5). The catalytic nucleophile (E374) and the catalytic acid/base (E163) residues of ORF1119 were conserved with other GH1 enzymes^26,27^. We prepared the recombinant BglH from *E. coli* cells, tested its specificity using *p*NP substrates, and found that BglH does not hydrolyze *p*NP-β-D-PvGal (Table 2). We further analyzed BglC, a closer homolog of ORF1119 from *Clostridium saccharoperbutylacetonicum* (Figs 4 and 5). Using the same methods, we revealed that BglC exhibits PvGal-ase activity similar to that of ORF1119 protein (Table 2).

**Table 2:** Substrate specificity of ORF1119, BglC and BglH.

Substrate	ORF1119^a^	BglC^a^	BglH^a^
*p*NP-α-Gal	−	−	−
*p*NP-β-Gal	++	++	+
*p*NP-α-Man	−	−	−
*p*NP-β-Man	−	−	+
*p*NP-β-Glc	+	+	+
*p*NP-β-GlcNAc	−	−	−
*p*NP-β-Lac	−	−	−
*p*NP-β-GalNAc	−	−	−
*p*NP-α-Xyl	−	−	−
*p*NP-β-Xyl	++	++	++
*p*NP-β-PvGal	++	++	−
*p*NP-β-PvLac	−	−	−

^a^Enzymatic activity is indicated as follows: −, none; +, weak; ++, strong.

### Crystal structure of ORF1119 PvGal-ase

**Figure 6:**
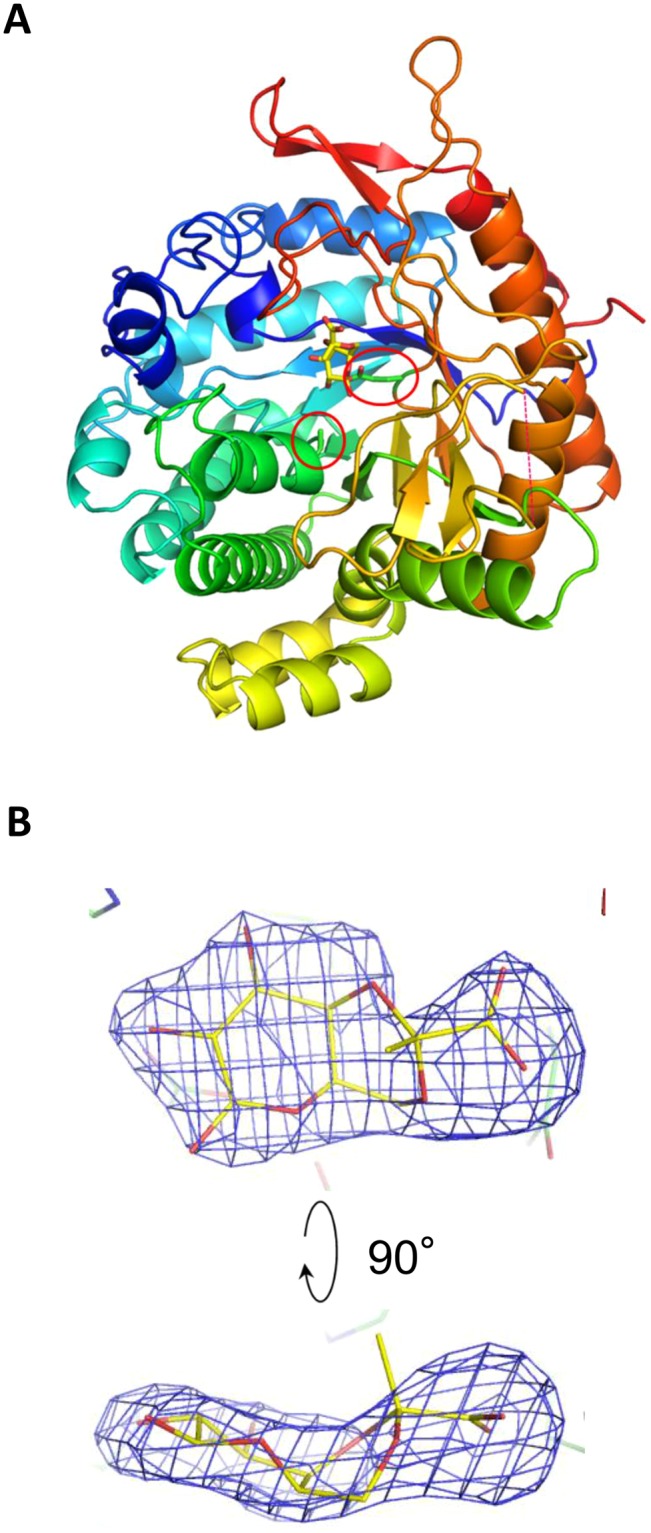
Crystal structure of ORF1119. (**A**) Overall structure of ORF1119 (E163A mutant) complexed with PvGal (yellow). Chain A is shown in rainbow colors. The side chains of the catalytic residues (nucleophile E347 and acid/base mutant E163A) are shown as sticks and highlighted by red circles. The disordered region is shown as a red dotted line. (**B**) *m*F_o_-*D*F_c_ electron density map (4.0σ, blue mesh) of PvGal in the complex structure.

Because ORF1119 protein exhibited hydrolase activity toward both *p*NP-β-D-PvGal and *p*NP-β-D-Gal, we investigated which amino acid residues are responsible for the recognition of these substrates. The crystal structure of ORF1119 was determined by molecular replacement using the structure of *Halothermothrix orenii* BGL^28,29^ (HoBGL, PDB code 3TA9) as a search model. Structures of the ligand-free form and PvGal-bound form were determined at 2.5 Å and 2.45 Å resolution, respectively (Table 3). The complex crystal was prepared by co-crystallization using the catalytic acid/base residue mutant E163A and *p*NP-β-D-PvGal. The crystals belonged to space group *P*322_1_ and contained two protein molecules in the asymmetric unit. Part of the structure was disordered (residues 311–320 in chain A of the PvGal complex) and was not included in the final model (red dotted line in Fig. 6A). Because the structures of all four chains (chains A and B in both the ligand-free and complex forms) were almost the same (root mean square deviation for Cα atoms, <0.6 Å), below we describe chain A of the PvGal-bound form. The overall structure of ORF1119 was similar to those of other GH1 enzymes, which have a typical (β/α)_8_ barrel fold (Fig. 6A). The active site is located at the bottom of the pocket formed by loops of the barrel, a characteristic of most GH1 enzymes.

**Table 3:** Statistics for X-ray crystallography.

	Ligand free (WT)	PvGal complex (E163A)
**Data collection**		
PDB entry	5YHS	5YIF
Beam line	Spring-8 BL26B1	PF BL17A
Wavelength (Å)	1.0000	0.9800
Space group	*P*3_2_21	*P*3_2_21
Unit cell (Å)	*a* = *b* = 113.4 *c* = 148.7	*a* = *b* = 114.8 *c* = 150.4
Resolution (Å)^a^	50.00–2.50 (2.54–2.50)	50.00–2.45 (2.49–2.45)
Total reflections	357,660	226,381
Unique reflections	39,354	42,682
Completeness (%)^a^	100 (100)	99.9 (99.9)
Redundancy^a^	9.1 (9.1)	5.3 (5.1)
Mean *I*/*σ* (*I*)^a^	19.5 (1.7)	12.1 (1.6)
*R*_merge_ (%)^a^	7.3 (98.9)	8.1(85.5)
CC_1/2_^a^	(0.777)	(0.621)
**Refinement**		
Resolution (Å)	98.70-2.50	99.40-2.45
No. of reflections	37,262	40,594
*R*/*R*_free_ (%)	21.9/27.6	20.6/27.0
No. of atoms	7,462	7,785
RMSD from ideal values		
Bond lengths (Å)	0.0126	0.0141
Bond angles (°)	1.643	1.737
Ramachandran plot (%)		
Favored	93.3	95.9
Allowed	6.1	3.4
Outlier	0.6	0.7

^a^Values in parentheses correspond to the highest resolution shell.

### Active site architecture

**Figure 7:**
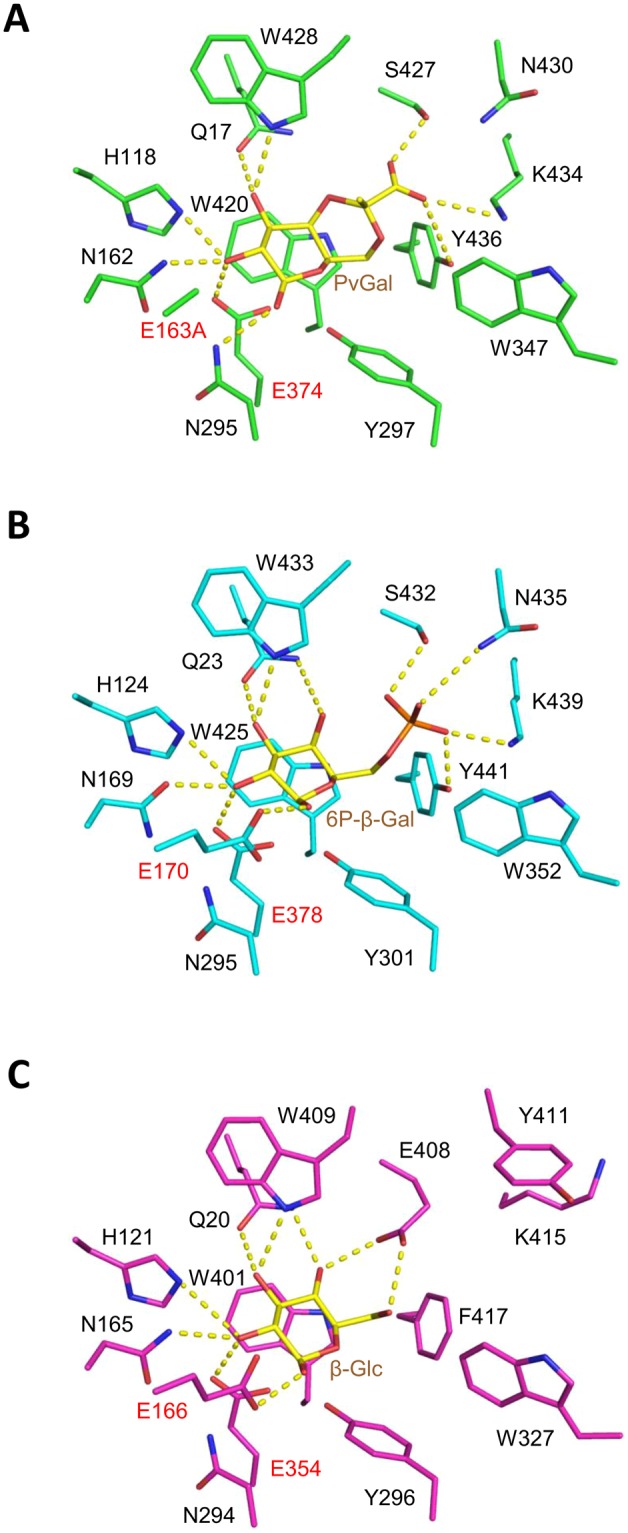
Active-site structures of three GH1 enzymes. (**A**) ORF1119 (E163A mutant, green) complexed with PvGal (yellow), (**B**) Gan1D (cyan) complexed with 6-phospho-β-galactose (yellow), and (**C**) HoBGL (magenta) complexed with β-glucose (yellow). The two catalytic residues are labeled in red. Hydrogen bonds are shown as yellow dotted lines.

In the complex structure, clear electron density is observed for the PvGal ligand, apart from the methyl group in the pyruvate moiety of PvGal (Fig. 6B). Although the complex structure was obtained by co-crystallization with *p*NP-β-D-PvGal, electron density of the *p*NP moiety is not observed in the structure, probably due to slow hydrolysis of the substrate during crystal growth. It was shown that a catalytic acid residue mutant (E170G) of β-glucosidase from *Agrobacterium faecalis* exhibited weak but significant remaining activity (*k*_cat_ = 0.015 s^–1^) toward *p*NP-β-D-glucoside, which has a “good” leaving group (alcohol of low p*K*_a_)^27^. The remaining activity of the catalytic acid/base residue mutant was considered to be enough for producing released PvGal during the long time scale of protein crystallization (typically 1–4 weeks in this study). The catalytic residues of ORF1119, E374 (nucleophile) and E163A (acid-base mutant) are in close proximity to the anomeric C1 and O1 atoms of PvGal, respectively (Fig. 7A). The O-2 and O-3 hydroxyl groups of PvGal are extensively recognized, forming one or more direct hydrogen bonds with the side chains of Q17, H118, N162 and W428. The carboxyl group of pyruvate forms hydrogen bonds with S427, K434 and Y436. W420 forms a stacking interaction with the sugar ring. Figure 7B,C show the active site structures of two GH1 enzymes: 6-phospho-β-glucosidase/galactosidase Gan1D from *Geobacillus stearothermophilus*^30^ (PDB code 4ZEN, sequence identity 44.6%), and β-glucosidase HoBGL from *Halothermothrix orenii*^28,29^ (PDB code 4PTX, sequence identity 39.1%). As shown in Fig. 7, the three-dimensional position of the active site residues (E374 and E163) of ORF1119 were also conserved with other GH1 enzymes. Almost all of the residues that recognize the sugar, except for residues around the O-6 hydroxyl group, are conserved in these enzymes. Residues around the O-6 hydroxyl group are conserved between ORF1119 and Gan1D, both of which have substrate preference toward sugars carrying a negatively charged group near the O-6 atom. In HoBGL, three different residues (E408, Y411 and F417) are located around the O-6 hydroxyl group, and the side chain conformation of K415 is different from that of K434 in ORF1119. These results indicate that the four residues in this region (S427, N430, K434 and Y436) of ORF1119 seem to be involved in binding to the pyruvate moiety in ORF1119.

### Mutational analysis

We constructed seven mutant proteins in which the four residues identified above were substituted with either the corresponding amino acid of HoBGL or Ala (S427A, S427E, N430A, N430Y, K434A, Y436A and Y436F). Table 4 lists the kinetic parameters of the wild-type ORF1119 and mutant enzymes toward *p*NP-β-D-PvGal. As expected, the activity of the two S427 mutant enzymes (S427A and S427E) toward *p*NP-β-D-PvGal was significantly reduced owing to the increased *K*_m_ value. In particular, the *k*_cat_/*K*_m_ value of S427E was reduced 200-fold relative to that of the wild-type enzyme, indicating that the elongated side chain of Glu probably leads to steric hindrance with the large pyruvylated group of PvGal. The Y436A enzyme also showed reduced activity (approximately 10-fold in *k*_cat_/*K*_m_) toward *p*NP-β-D-PvGal owing to the reduced *k*_cat_ value, while substitution of Y436 with a residue of a similar-sized side chain (Y436F) did not largely affect the activity. This result suggests that the large aromatic side chain of Y436 supports recognition of the pyruvylated group. Substitution of the other residues (N430 and K434) led to similar or slightly increased activity toward *p*NP-β-D-PvGal, suggesting that these residues do not play important roles in substrate recognition.

**Table 4:** Kinetic parameters of wild-type and mutant ORF1119 enzymes toward *p*NP-substrates.

Enzyme	pNP-β-D-PvGal			pNP-β-D-Gal		
	K_m_	k_cat_	k_cat_/K_m_	K_m_	k_cat_	k_cat_/K_m_
WT	0.81 ± 0.14	5.2 ± 0.2	6.3 ± 0.7	3.3 ± 0.4	0.20 ± 0.005	0.060 ± 0.004
S427A	1.92 ± 0.45	4.4 ± 0.3	2.3 ± 0.3	4.1 ± 0.4	0.09 ± 0.002	0.023 ± 0.001
S427E	—	—	0.025	0.89 ± 0.09	0.13 ± 0.002	0.149 ± 0.011
N430A	0.90 ± 0.16	7.0 ± 0.2	7.7 ± 0.9	4.0 ± 0.7	0.14 ± 0.006	0.035 ± 0.004
N430Y	0.37 ± 0.08	7.7 ± 0.2	21 ± 3	17 ± 1	0.65 ± 0.021	0.038 ± 0.002
K434A	0.73 ± 0.12	8.8 ± 0.2	12 ± 1	4.6 ± 0.3	0.25 ± 0.005	0.055 ± 0.002
Y436A	0.80 ± 0.18	0.68 ± 0.03	0.86 ± 0.1	0.8 ± 0.1	0.05 ± 0.001	0.061 ± 0.007
Y436F	0.53 ± 0.09	6.5 ± 0.2	12 ± 2	2.8 ± 0.3	0.20 ± 0.005	0.070 ± 0.005
**Enzyme**	***p*NP-β-D-Glc**			***p*NP-β-D-Fuc**		
	***K***_**m**_	***k***_**cat**_	***k***_**cat**_**/*K***_**m**_	***K***_**m**_	***k***_**cat**_	***k***_**cat**_**/*K***_**m**_
WT	7.4 ± 0.4	0.10 ± 0.002	0.014 ± 0.0005	1.9 ± 0.1	1.6 ± 0.02	0.83 ± 0.03
S427A	8.1 ± 0.6	0.09 ± 0.002	0.011 ± 0.0005	1.3 ± 0.08	0.64 ± 0.008	0.49 ± 0.02
S427E	0.46 ± 0.09	0.05 ± 0.001	0.098 ± 0.014	0.47 ± 0.06	0.91 ± 0.02	2.0 ± 0.2
N430A	5.3 ± 0.5	0.07 ± 0.002	0.013 ± 0.0007	1.5 ± 0.1	0.71 ± 0.01	0.48 ± 0.02
N430Y	16 ± 2	0.55 ± 0.02	0.034 ± 0.002	2.8 ± 0.3	4.6 ± 0.1	1.6 ± 0.09
K434A	12 ± 1	0.17 ± 0.005	0.014 ± 0.0007	0.81 ± 0.06	0.88 ± 0.01	1.1 ± 0.06
Y436A	33 ± 5	0.04 ± 0.003	0.001 ± 0.0001	2.3 ± 0.2	0.18 ± 0.003	0.077 ± 0.004
Y436F	5.4 ± 0.5	0.22 ± 0.006	0.040 ± 0.002	0.67 ± 0.07	0.66 ± 0.01	0.98 ± 0.07

We also measured the kinetic parameters of enzyme activity toward three other substrates, *p*NP-β-D-Gal (without the pyruvylated group), *p*NP-β-D-Glc (4-epimer of Gal), and *p*NP-β-D-Fuc (6-deoxy variant of Gal). Wild-type ORF1119 showed relatively higher activity toward *p*NP-β-D-Fuc as compared with *p*NP-β-D-Gal or *p*NP-β-D-Glc, indicating that the enzyme prefers a hydrophobic group at the C-6 position. The S427E mutant enzyme exhibited higher activity toward these three substrates (2.4 ~ 7.0-fold increase in the *k*_cat_/*K*_m_) as compared with the wild-type enzyme. The longer side chain of Glu might alter the active site so that it is suitable for non-pyruvylated substrates. Other mutations also slightly affected the activity toward non-pyruvylated substrates, but the changes in *k*_cat_/*K*_m_ values were less than those observed for the S427E mutant enzyme. Collectively, these results indicate that S427 is the most crucial residue for the recognition of PvGal moiety.

### ORF1119 protein releases PvGal from fission yeast galactomannan

**Figure 8:**
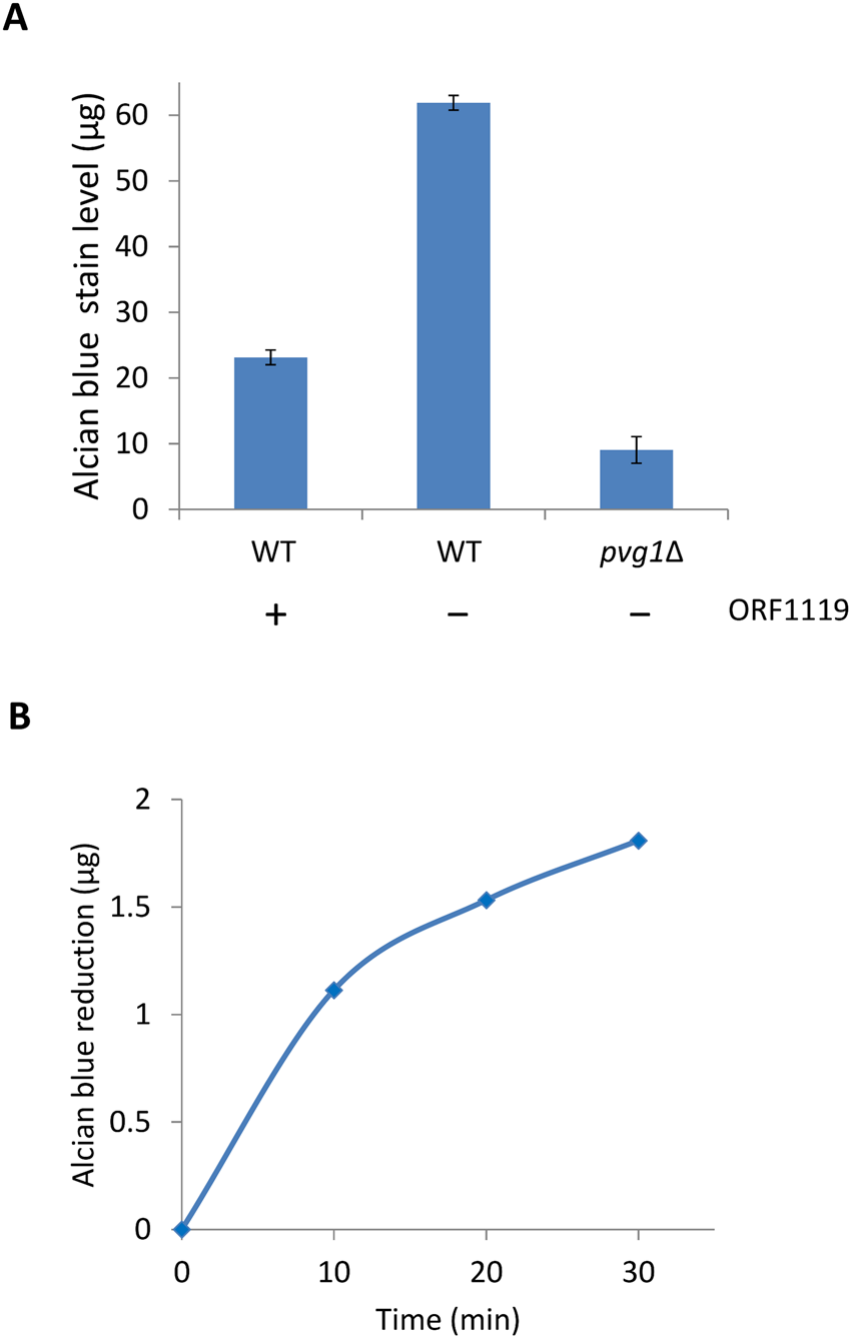
Analysis of ORF1119 PvGal-ase activity toward fission yeast galactomannan. (**A**) Level of extracellular negatively charged glycans. *S. pombe* WT strain in the presence or absence of recombinant ORF1119 PvGal-ase was stained with alcian blue. *S. pombe pvg1*Δ strain was used as a negative control. (**B**) Time course of the reduction in alcian blue staining of *S. pombe* WT strain incubated with recombinant ORF1119 PvGal-ase.

Finally, we tested whether ORF1119 PvGal-ase can catalyze not only the artificial substrate *p*NP-β-D-PvGal but also a natural PvGal-containing oligosaccharide. PvGal is present in glycans on the cell surface of *S. pombe*^6,7^. We stained the cells with alcian blue, which attaches to negatively charged oligosaccharides, including the pyruvate moiety of PvGal. The addition of ORF1119 to *S. pombe* cells significantly reduced the level of alcian blue staining (Fig. 8A). We confirmed that the reduction in alcian blue staining was time-dependent, suggesting that the ORF1119 reaction occurred enzymatically and that ORF1119 can hydrolyze a natural PvGal-containing oligosaccharide (Fig. 8B).

## Discussion

In this study, we isolated a *Bacillus* strain that harbors a PvGal-ase encoded by ORF1119. This protein belongs to the GH1 family, which generally has broad substrate specificity. Indeed, ORF1119 protein exhibited hydrolytic activity not only toward *p*NP-β-PvGal but also weakly toward *p*NP-β-Gal, suggesting that it catalyzes hydrolysis of these substrates at the same active site but discriminate these substrates. Moreover, ORF1119 protein could not hydrolyze *p*NP-β-Lac or *p*NP-β-PvLac, both of which include a Glc residue between the Gal and *p*NP moieties, suggesting that ORF1119 protein exclusively recognizes a galactose-type monosaccharide group at subsite −1 and does not accept a Glc moiety at subsite +1.

Our BLAST search revealed that ORF1119 protein-like PvGal-ases are present in a variety of microorganisms (Fig. 4). Among them, we further examined two ORF1119 homologs: the putative β-D-glucosidase BglC from *Clostridium* and the aryl-phospho-β-D-glucosidase BglH from *Bacillus*. Although the catalytic residues (E163 and E374 in ORF1119) are conserved among these homologs, BglH did not exhibit PvGal-ase activity toward *p*NP-β-PvGal as a substrate, suggesting that ORF1119 homologs contain similar active sites but have different substrate specificities.

On the basis of our structural and mutational analyses, we propose that S427 is responsible for recognition of PvGal, and the aromatic side chain of Y436 supports this recognition. In contrast, N430 and K434 are not crucial residues. In addition, we confirmed that ORF1119 PvGal-ase catalyzes not only artificial *p*NP substrates but also the natural substrate *S. pombe* galactomannan. In the genome of *S. pombe*, there is no obvious homolog of ORF1119. Therefore, it is currently hard to predict how *S. pombe* galactomannan is metabolized. Previously, we generated pyruvylated human-type complex glycopeptides by using a Pvg1p^H168C^ mutant that can attach Pv to the terminal β-1,4-linked Gal residue of human-type complex glycopeptide^20^. Thus, we also tested whether ORF1119 PvGal-ase can cleave PvGal from pyruvylated human-type complex glycopeptide; in this case, however, we did not detect enzymatic activity (data not shown). This might be because ORF1119 PvGal-ase can catalyze β-1,3-linked PvGal in *S. pombe* galactomannan but cannot cleave β-1,4-linked PvGal in human-type complex glycopeptide. Further molecular dissection of the substrate recognition mechanisms of ORF1119 PvGal-ase will be required to engineer an enzyme that can catalyze pyruvylated human-type complex oligosaccharides.

In summary, we have characterized a novel PvGal-ase encoded by ORF1119 in *Bacillus* strain HMA207. Given that PvGal may exist in oligosaccharide structures of versatile organisms, more PvGal-ases are likely to be identified in addition to ORF1119 protein and BglC. Because Pv has similar features to mammalian sialic acid, PvGal-catalyzing enzymes would be beneficial for novel glycoengineering to produce new oligosaccharides with sialic acid-mimicking property.

## Electronic supplementary material

Supplementary information
