# Author Correction: Identification and characterization of a novel β-D-galactosidase that releases pyruvylated galactose

**DOI:** 10.1038/s41598-020-60002-9

**Published:** 2020-02-13

**Authors:** Yujiro Higuchi, Hitomi Matsufuji, Masanari Tanuma, Takatoshi Arakawa, Kazuki Mori, Chihaya Yamada, Risa Shofia, Emiko Matsunaga, Kosuke Tashiro, Shinya Fushinobu, Kaoru Takegawa

**Affiliations:** 1 Department of Bioscience and Biotechnology, Faculty of Agriculture, Kyushu University, 6-10-1 Hakozaki, Fukuoka, 812-8581 Japan; 2 Department of Biotechnology, The University of Tokyo, 1-1-1 Yayoi, Bunkyo-ku, Tokyo 113-8657 Japan

Correction to: *Scientific Reports* 10.1038/s41598-018-30508-4, published online 13 August 2018

This Article contains typographical errors in the Materials and Methods section under subheading ‘Accession Numbers’.

“The nucleotide sequences of the ORF1119, ORF4395 and ORF4971 genes have been deposited in the DDBJ/EMBL/GenBank under the accession nos. LC306881, LC306883 and LC306885, respectively.”

should read:

“The nucleotide sequences of the ORF1119, ORF4395 and ORF4971 genes have been deposited in the DDBJ/EMBL/GenBank under the accession nos. LC317050, LC317052 and LC317053, respectively.”

