# Supplementary material for: Identification and characterization of a novel β-D-galactosidase that releases pyruvylated galactose
Source: Sci Rep. 2018 Aug 13;8:12013. doi: 10.1038/s41598-018-30508-4 (PMC6090015; doi:10.1038/s41598-018-30508-4)
Supplement: Supplementary file 1 — Supplementary information [file 41598_2018_30508_MOESM1_ESM.pdf]

Supplementary Information

Identification and characterization of a novel β-D-galactosidase that releases pyruvylated galactose

Yujiro Higuchi[1], Hitomi Matsufuji[1], Masanari Tanuma[2], Takatoshi Arakawa[2], Kazuki Mori[1], Chihaya Yamada[2], Risa Shofia[1], Emiko Matsunaga[1], Kosuke Tashiro[1], Shinya Fushinobu[2], Kaoru Takegawa[1,*]

[1]Department of Bioscience and Biotechnology, Faculty of Agriculture, Kyushu University, 6-10-1 Hakozaki, Fukuoka 812-8581, Japan

[2]Department of Biotechnology, The University of Tokyo, 1-1-1 Yayoi, Bunkyo-ku, Tokyo 113-8657, Japan.

[*]Corresponding author. Tel/Fax: +81 92 642 2851, E-mail address: takegawa@agr.kyushu-u.ac.jp (K. Takegawa)

Number of words: Abstract, 200; Main, 5005.

Number of figures: Main, 8; Supplementary, 1.

Number of tables: Main, 4; Supplementary, 1.

Key words: *Bacillus* species, β-D-galactosidase, galactomannan, pyruvylated galactose

Running title: Pyruvylated-galactose-releasing galactosidase

Supplementary figure legend

Supplementary Figure S1. Image of the original SDS-PAGE gel that was cropped and used in Figure 2A.

Shown is the full image of the original SDS-PAGE gel that was cropped and used in Figure 2A. Lane M, molecular weight markers; lane 1, supernatant; lane 2, pellet; lane 3, flow through; lane 4, wash; lanes 5-11, elution.

Supplementary Figure S1

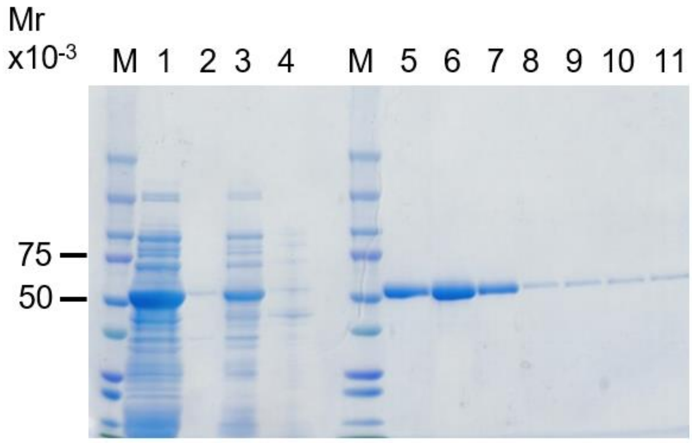

Supplementary Table 1. Primers used in this study.

Target	Sequence (5’ to 3’; upper, forward; lower, reverse)
16S rRNA gene	AGAGTTTGATCMTGGCTCAG (M: A or C)
	GGYTACCTTGTTACGACTT (Y: A, T or C)
pET-32b	CACCACCACCACCACCACTGAGAT
	ATGTATATCTCCTTCTTAAAGTTA
ORF1119	GAAGGAGATATACATATGAAGTTTCCACATGATTTT
	GTGGTGGTGGTGGTGTCATTATAACTCTTCCCCTCTC
ORF1119	GAAGGAGATATACATATGAAGTTTCCACATGATTTT
(for His tag)	GTGGTGGTGGTGGTGTAACTCTTCCCCTCTC
ORF4395	GAAGGAGATATACATCATGACTGGAATTAAAATTGCT
	GTGGTGGTGGTGGTGTCATTACTTCTCTACCTTTTTAAA
ORF4971	GAAGGAGATATACATCATGAAGGATTGGCATAAAAG
	GTGGTGGTGGTGGTGTCATTATTTCGTATATCGATACAC
BglC	GAAGGAGATATACATATGAAATTTTCTAAAGACTTTCTATTTGGAG
	GTGGTGGTGGTGGTGAAGATTTTCGCCTCTCTCTTC
BglH	GAAGGAGATATACATATGAGTTCAAATGAAAAACG
	GTGGTGGTGGTGGTGGAGACTCTCTCCGTTTG
H118A	TTATATgctTGGGATTTACCATTACCGTTAGAAAAAG
	ATCCCAagcATATAAAGTGACAAACGGCACAATC
E163A	TTTAATgctACCGTAATGTTTTGTGGATTAGG
	TACGGTagcATTAAAAGTAATCCAATGCTTAAC
S427A	CTTTTAgctTGGTTAAATGGATATAAA
	AGTCACTAACTAGAAAATcgaACCAAT

S427E	CTTTTAgaaTGGTTAAATGGATATAAA
	AGTCACTAACTAGAAAATcttACCAAT
N430A	TGGTTAgctGGATATAAAAAGCAATAT
	CTAGAAAATTCAACCAATcgaCCTATA
N430Y	TGGTTAtatGGATATAAAAAGCAATAT
	CTAGAAAATTCAACCAATataCCTATA
K434A	TATAAAgctCAATATGGCTTTATTTTT
	ACCAATTTACCTATATTTcgaGTTATA
Y436A	AAGCAAgctGGCTTTATTTTTGTCGAT
	TTACCTATATTTTTCGTTcgaCCGAAA
Y436F	AAGCAAtttGGCTTTATTTTTGTCGAT
	TTACCTATATTTTTCGTTaaaCCGAAA

Lower case characters represent nucleotides where point mutations were introduced.

The nucleotide sequence of ORF1119 was used as a template for all point mutants.
